# Protocol for integrating vessel networks and environmental matching to inform cost-effective archipelago biosecurity planning and management

**DOI:** 10.1016/j.xpro.2026.104797

**Published:** 2026-08-24

**Authors:** Chi T.U. Le, Chad L. Hewitt, Marnie L. Campbell

**Affiliations:** 1 School of Science, Edith Cowan University, 270 Joondalup Drive, Joondalup Western Australia, Australia; 2 Centre for One Biosecurity Research, Analysis and Synthesis, Lincoln University, PO Box 85084, Lincoln Christchurch 7647, New Zealand; 3 University of Aberdeen – Mumbai, Chromium Andheri East, Jogeshwari - Vikhroli Link Rd, Powai, Mumbai, Maharashtra 400072, India

**Keywords:** Earth sciences, Environmental sciences, Systems biology

## Abstract

Inter-island vessel networks support economic activity but also enable secondary spread of non-native species. By integrating vessel-movement data, network analysis, and environmental matching, we present a protocol for generating a cost-effective biosecurity decision-support tool by assessing both the relative economic importance and the relative risks of species spread within a vessel traffic system. We outline steps for data curation, network construction, and sea surface temperature-based environmental similarity calculation. We then detail procedures for the translation of outputs into management recommendations.

For complete details on the use and execution of this protocol, please refer to Campbell et al.^1^

## Highlights

• A workflow for supporting biosecurity management based on vessel movement data
• Steps to integrate environmental matching into vessel networks for risk assessment
• Steps for ranking islands and communities for biosecurity management and planning

## Abstract (editor highlights)

Publisher’s note: Undertaking any experimental protocol requires adherence to local institutional guidelines for laboratory safety and ethics.

## Graphical abstract

**Figure:**
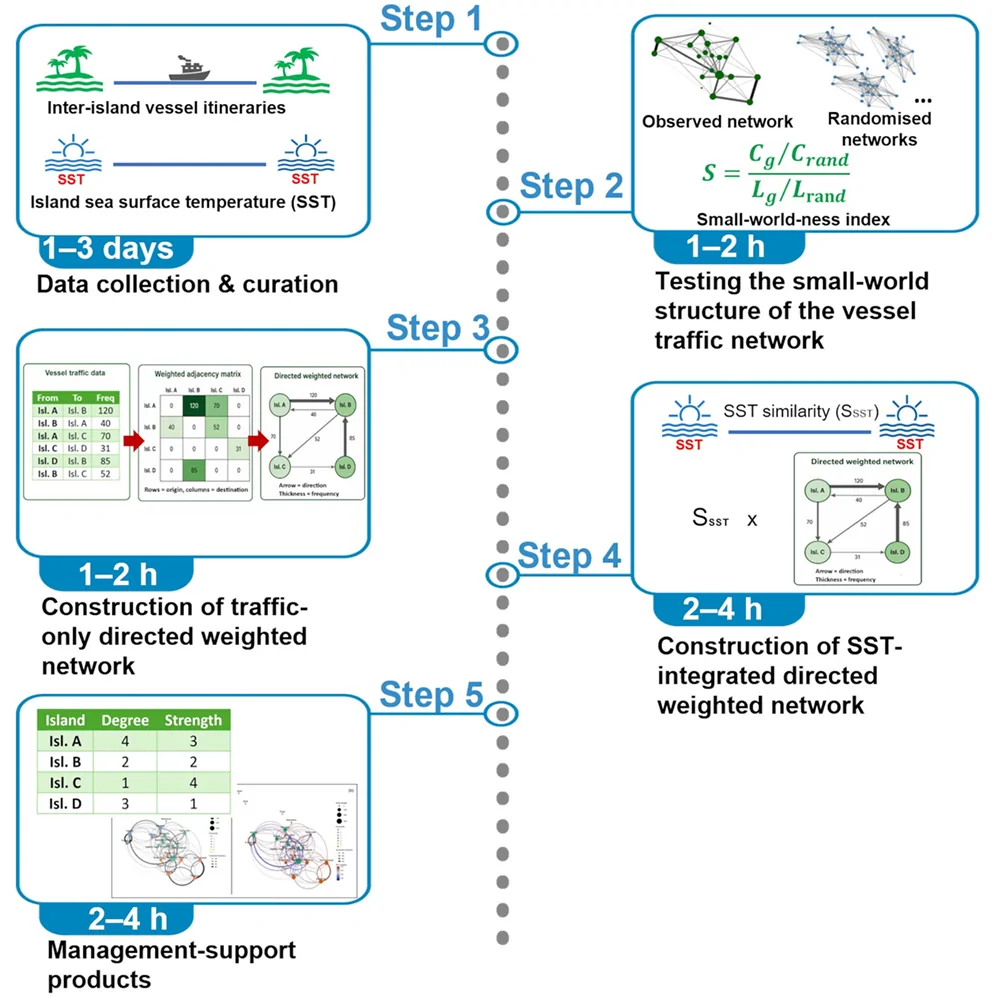

## Before you begin

This protocol guides the analysis of tourist small-vessel movement among islands using network analysis, as demonstrated in a case study of the Galápagos archipelago, Ecuador. The workflow combines these vessel-movement data with island-level sea surface temperature (SST) to quantify connectivity, identify communities, and assess island- and community-level relative vulnerability relevant to economic importance and the spread of non-native species. The resulting network outputs are used to develop surveillance priorities, route-management options, and biosecurity zoning recommendations that balance biosecurity and economic outcomes.

In the associated Galápagos case study,^1^ the authors define islands as analytic nodes and represent shipping routes as directed edges weighted by trip frequency. The authors treat SST as an environmental filter because sea temperature strongly influences marine species’ growth, distribution, and survival,^2^^,^^3^^,^^4^^,^^5^^,^^6^ and the region spans a broad SST gradient.^7^ The study window is from February 2015 to January 2016, and monthly SST values are derived from the mean SST recorded on the first day of each month. When applying this protocol to other systems, users should ensure that node and edge definitions, environmental variables, and the study window are context-appropriate and internally consistent.

### Innovation

This protocol advances biosecurity decision-support by integrating vessel-movement connectivity with environmental matching in a unified analytical workflow. By incorporating a Gaussian-kernel SST similarity term into edge weights, the approach captures both the anthropogenic importance of shipping routes and the environmental suitability that influences species survival. This integration enables more transparent, biologically informed, and cost-effective prioritisation of surveillance, management interventions, and biosecurity zoning across archipelagos and other connected systems.

### Institutional permissions

Access to vessel-movement, itinerary, operator, or management data may be restricted in some jurisdictions. Ensure that all data collection and use comply with relevant institutional and national regulations. Readers must obtain the necessary permissions from vessel operators, port authorities, tourism agencies, or protected-area managers before accessing, compiling, or sharing any restricted datasets.

### Curation of tourist small-vessel movement data

**Timing: 1–3 days**

This step guides the collection and curation of tourist vessel-traffic data from publicly available and authorised sources.

***Note:*** The timing for vessel-movement data collection is indicative. Where data access requires permissions, allow for additional time.

1. Define the scope and analytical unit for the network.

***Note:*** In the associated study, we defined 23 islands of the Galápagos archipelago as nodes, and represented directed edges as ship routes between pairs of islands weighted by trip frequency.

2. Collect the annual scheduled tourist-vessel traffic from publicly available tourist websites.
3. Extract departure and destination islands and the annual trip frequency for each route across the 12-month study period.
4. Curate vessel-movement data:
  a. Enter the data into an Excel spreadsheet as an *edge list* with three columns: *from* (departure island), *to* (destination island), and *frequency* (annual number of trips).
  b. Save the cleaned *edge list* in a machine-readable format (e.g., .csv).

***Optional:*** Collect geographic coordinates (latitude and longitude) for each island and assign them to the corresponding *from-to* pairs to support later graph visualisation that reflects the spatial layout of the archipelago.

***Note:*** In the associated study, only tourist small-vessel movement traffic was included due to data limitations. Incorporating other vessel types (e.g., cargo, recreational, liveaboard tourist), where available, will provide a more comprehensive assessment of ship-mediated biosecurity risk.

### Curation of environmental (SST) data

**Timing: 3–4 h**

5. Collect monthly SST data across the 12-month study period for all primary tourist locations and ports.

***Note:*** In the associated study, SST data were obtained from the State of the Oceans (SOTO) dataset (https://www.earthdata.nasa.gov/data/tools/soto) and extracted from the Group for High Resolution Sea Surface Temperature (GHRSST) daily 1 km MUR Level 4 SST values for each island location. Mean SST of the first day of each month was used in the case study because these data were consistently available across the islands.

6. Store monthly SST values for each island in an Excel spreadsheet.
7. Calculate the absolute SST difference for all possible island pairs for each month and store the results as a data table.

**CRITICAL:** Ensure that island pairs in the monthly SST difference dataset remain exactly aligned with the island set used in the vessel-movement *edge list***.**

***Note:*** Absolute SST difference is used to derive an undirected SST similarity metric between island pairs, representing route-level environmental matching. This metric is incorporated into the network to assess the relative risks of spreading species across shipping routes, rather than to quantify realised risks of species survival, establishment, or invasion success.

***Optional:*** Categorise the SST differences into 1°C bins if a descriptive summary is required.

### Installation of required software

8. Install the latest version of R appropriate for your operating system from https://www.r-project.org/, and follow the installation instructions.
9. Install RStudio (version 4.4.2 or the latest version) from https://posit.co/download/rstudio-desktop/ and complete the installation as instructed.
10. Install the required R packages for network analysis, visualisation, and data wrangling, including igraph,^8^ DirectedClustering,^9^ leidenAlg,^10^ ggraph,^11^ PMCMRplus,^12^ and tidyverse.^13^
11. Load all required packages using library() before beginning the analysis.

## Key resources table

**Table:** 

REAGENT or RESOURCE	SOURCE	IDENTIFIER
**Software and algorithms**		

R Statistical Software v4.4.2 or the latest version	R Core Team^14^	R v4.4.2, https://www.R-project.org/
RStudio v2024.9.1.394 or the latest version	Posit Team^15^	RStudio v2024.9.1.394, http://www.posit.co/
R package ‘igraph’	Csardi & Nepusz^8^	‘igraph’ R package version 2.1.4, https://igraph.org
R package ‘DirectedClustering’	Clemente^9^	‘DirectedClustering’ R package version 1.0.0, https://CRAN.R-project.org/package=DirectedClustering
R package ‘leidenAlg’	Kharchenko et al.^10^	‘leidenAlg’ R package version 1.1.5, https://CRAN.R-project.org/package=leidenAlg
R package ‘ggraph’	Pederson^11^	‘ggraph’ R package version 2.2.1, https://CRAN.R-project.org/package=ggraph
R package ‘PMCMRplus’	Pohlert^12^	PMCMRplus R package version 1.9.12, https://www.rdocumentation.org/packages/PMCMRplus/versions/1.9.12
R package ‘tidyverse’	Wickham et al.^13^	‘tidyverse’ R package version 2.0.0, https://doi.org/10.32614/CRAN.package.tidyverse

**Deposited data**		

Tourist vessel scheduled itinerary	Tourist websites	N/A
Sea surface temperature	State of the Oceans	https://www.earthdata.nasa.gov/data/tools/soto

## Step-by-step method details

### Testing small-world structure in the tourist vessel network

**Timing: 1–2 h**

This step evaluates whether the tourist-vessel system exhibits small-world structure by constructing an undirected weighted network and comparing its clustering coefficient and average path length with those of randomised networks.

1. Build an undirected weighted network.
  a. Import the vessel-traffic *edge list* into R and store it as a data frame.
  b. Store the unique island names in a separate data frame.
  c. Construct an undirected weighted network with islands as vertices and the connections in the *edge list* as edges.
  d. Simplify the graph to remove multiple edges and loops, and define edge weight as the sum of trip frequency between the corresponding island pair.
2. Calculate the small-world-ness index S following Humphries & Gurney.^16^
  a. Compute the global clustering coefficient (C_g_) of the observed network.
  b. Compute the observed weighted average path length (L_g_).
  c. Simulate 1,000 random networks that match the observed network in the number of nodes and edges, drawing edge weights from the observed weight distribution.
  d. Calculate the mean clustering coefficient (C_rand_) and mean path length (L_rand_) across the random networks.
    ***Optional:*** Calculate *p*-values to examine if C_g_ and L_g_ differ significantly from the corresponding metrics of random networks.
  e. Calculate the small-world-ness index using:
    $$S=\frac{C_{g}∕C_{rand}}{L_{g}∕L_{\mathrm{ran}d}}$$
  f. Interpret S > 1 as evidence of small-world structure.
    **CRITICAL:** Do not calculate path lengths using trip frequency as the edge weight. Invert weights so that they represent distance between node pairs for both observed and null-model analyses.
    ***Optional:*** Detect Louvain communities in the weighted network and compare network modularity between the observed and randomised networks for reporting.
    ***Note:*** See problem 1 if an error message occurs during the computation of mean path lengths.

### Build the directed weighted network from tourist vessel traffic

**Timing: 1–2 h**

This step describes how to construct the directed weighted tourist-vessel network used for traffic-only assessments of ship-mediated risk.

3. Create a directed weighted network from the curated vessel traffic. Use the edge list imported in earlier steps to construct a directed network in which islands are nodes and edges represent the direction and frequency of tourist vessel trips.
4. Calculate node-level network metrics relevant to biosecurity risk and shipping importance. Compute node out-degree, out-strength, betweenness, and out-closeness.

***Note:*** In a biosecurity context, node out-degree represents the number of outgoing connections, out-strength reflects the total weighted flow to other islands, betweenness indicates how often an island lies on shortest paths between others, and out-closeness reflects how easily an infection could spread from that island.

5. Rank islands by each node metric and export the ranking tables.
6. Detect directed weighted communities using the Leiden algorithm.^17^

***Note:*** Communities are useful because species introduced to one member island are more likely to spread within the same community than to islands outside it.

***Optional:*** Test the robustness of identified communities following the recommendations of Fortunato and Hric.^18^^,^^19^

7. Calculate community-level vulnerability metrics.

***Note:*** For each community, compute community size, number of bridges, internal flow, total inflow, total outflow, mean out-closeness, and mean betweenness.

***Note:*** In this protocol, a ‘bridge’ is defined as an inter-community edge, i.e., an edge connecting departure (source) and destination (target) nodes that belong to different communities, and ‘number of bridges’ refers to the total number of inter-community edges, regardless of their directions.

***Note:*** Higher community vulnerability metrics indicate that a community is more susceptible to receiving or spreading non-indigenous marine species once any member island becomes infected.

**CRITICAL:** Use trip frequency as edge weight when calculating strength-based metrics (e.g., node out-strength).

**CRITICAL:** Use the inverse of trip frequency when calculating distance-based metrics (e.g., out-closeness, betweenness) so that edge weights represent the distance between nodes.

***Optional:*** Calculate network modularity and compare it with the mean modularity of 1,000 randomised directed weighted networks that preserve the observed directed network topology, including the number of nodes, the number of edges, edge directions, and node-level in- and out-degrees, while weights are drawn from the observed trip frequency distribution.

***Note:*** See problem 2 and the associated Potential Solution.

### Compute SST similarity and identify the month with the highest similarity

**Timing: 1–2 h**

This step converts pairwise absolute SST differences into biologically meaningful SST similarity values and tests for monthly differences in SST similarity across the study region.

8. Calculate monthly SST similarity for every island pair, using the equation adapted from Seebens et al.^20^:

$$S_{\text{SST}}={\mathrm{e}}^{-\left(\frac{\Delta T^{2}}{2\sigma_{T}^{2}}\right)}$$

Where ΔT is the absolute SST difference between a pair of islands, and σ_T_ = 2 controls the rate at which similarity declines as ΔT increases.

***Note:*** The choice of σ_T_ = 2 has been used as a general scaling parameter applied across multiple species in shipping-mediated marine biovasion risk models (e.g., Seebens et al.,^20^^,^^21^ Saebi et al;^22^). However, it should not be interpreted as a universal physiological tolerance threshold. To assess the robustness of the monthly SST comparisons, recalculate SST similarity using different scaling parameters and repeat the comparisons for a sensitivity analysis. For species-specific analyses, select σ_T_ based on biological traits such as thermal tolerance.

***Note:*** S_SST_ values fall within (0, 1], with higher values indicating greater environmental similarity and potentially greater survival of introduced species.

9. Compare SST similarity among the months using a Friedman’s test.
10. If the Friedman’s test is significant, perform post-hoc Conover’s all-pairs comparison tests for Friedman-type ranked data with an appropriate *p*-value adjustment method.

***Note:*** Although the associated case study used the Kruskal-Wallis test as a simple rank-based comparison of monthly SST similarity, Friedman’s test is recommended in this protocol because it better accounts for the repeated-measures structure of the SST similarity data. Reanalysis of the associated case study using Friedman’s test led to the same qualitative interpretation.

11. Identify the month with the highest median SST similarity and retain that month’s matrix for the illustrative composite-network presentation.

***Note:*** The month with the highest SST similarity represents the period of greatest potential survival for introduced marine species, which corresponds to a precautionary worst-case scenario, and is therefore used for biosecurity-focused illustration.

### Build SST-integrated directed weighted networks

**Timing: 1–2 h**

This step integrates traffic frequency and SST similarity into composite edge weights to assess vessel-mediated biosecurity risks.

12. Merge the SST similarity dataset with the vessel-traffic edge list.
13. Create a composite weight for each island pair by multiplying the trip frequency by the monthly SST similarity for the corresponding pair.
14. Build the SST-integrated directed weighted network using the composite weights as edge weights.
15. Recalculate the same network statistics used for the traffic-only network (see Steps 4–7), including node-level metrics, community detection, and community-level vulnerability metrics.

### Translate network outputs into management-support products

**Timing: 1–2 h**

This step converts quantitative network outputs into island-level risk-ranking tables, visual products, and biosecurity-relevant interpretations for managers.

16. Generate ranked island tables based on node out-degree, out-strength, betweenness, and out-closeness for both the traffic-only and SST-integrated networks.
17. Generate ranked community tables based on community vulnerability metrics.
18. Produce network visualisations to support intuitive assessment of connectivity patterns and biosecurity risk.
  a. Create two graphical visualisations: one for the traffic-only vessel network and one for the SST-integrated vessel network.
  b. Tailor visual encodings to management priorities.

**Figure 1:**
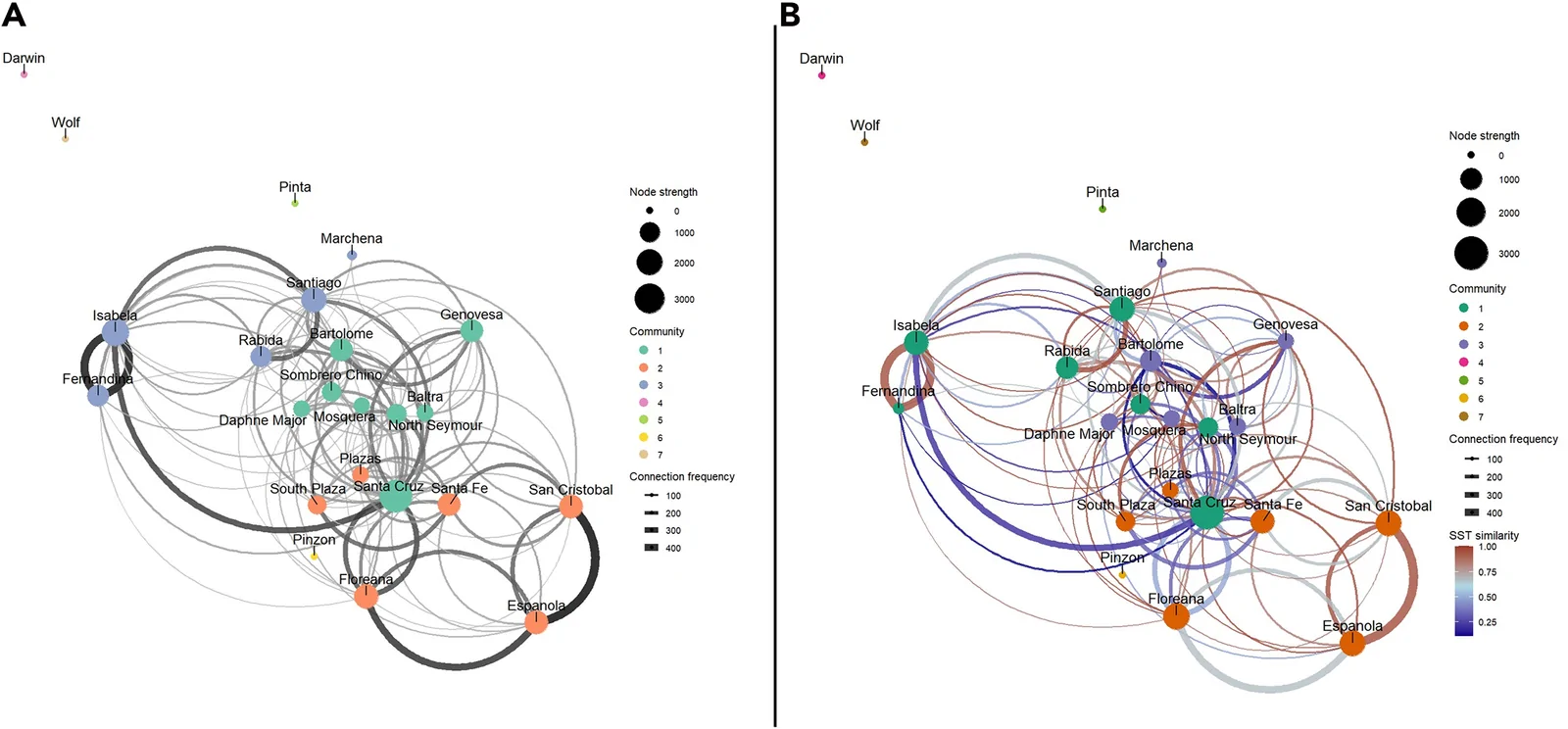
Management-support visualisations of the tourist vessel networks (adapted from Figures 1 & 2 in Campbell et al.^1^) (A) Traffic-only directed weighted network, and (B) Sea surface temperature (SST)-integrated directed weighted network.

***Note:*** Examples for visual encodings (see Figure 1) include sizing nodes by a relevant node-level metric (e.g., node out-strength), colouring node by community membership, scaling edge thickness by edge weight, and encoding edge colour by SST similarity (in the SST-integrated network).

19. Use the traffic-only network to assess both island shipping importance and ship-mediated biosecurity risk.
20. Use the SST-integrated network to assess ship-mediated biosecurity risk while incorporating environmental matching.

***Note:*** In the associated study, nodes are positioned according to the geographic locations of the islands to reflect the spatial structure of the archipelago, with minor adjustments in dense clusters to improve visual clarity.

## Expected outcomes

Users following this workflow obtain three key outputs, including: 1) ranked island tables that quantify and compare relative shipping importance and biosecurity risk among individual islands; 2) identified island communities ranked according to community-level vulnerability metrics; and 3) network visualisations that illustrate connectivity patterns and associated biosecurity risks. Highly ranked islands and communities, and high-risk connections (e.g., edges with high weights, important inter-community bridges) should be prioritised for management attention and efforts. As each metric represents an aspect of marine bioinvasion risk, these rankings can inform targeted planning and management strategies. Together, these products form a transparent and informative decision-support tool that supports evidence-based biosecurity planning and management.

## Limitations

The robustness of the outputs depends on the quality and completeness of the input data and on the assumptions used in constructing the networks. First, shipping importance and biosecurity relative risk are derived from vessel movement data; incomplete or inaccurate records may lead to under- or over-estimation of relative risks. Second, nodes are defined at the island level. While this scale is practical for management, environmental data may not capture within-island heterogeneity between monitoring stations, ports, or anchoring sites. Third, SST is the only environmental filter incorporated in the protocol. Although SST is relevant in the present case study, other environmental variables (e.g., salinity) and additional factors (e.g., inter-island distance, propagule pressure associated with vessel type and size) may also influence the likelihood of spreading non-native species. Where multiple variables, such as environmental conditions or vessel characteristics, are integrated, their potential interactions with bioinvasion risks or with each other should be considered. In species-specific cases, species distribution model (SDM) may provide a useful way to represent the combined influence of relevant variables, where suitable data and modelling techniques are available. Finally, because edge weight is defined as the product of trip frequency and SST similarity, it represents a proxy for propagule pressure and habitat suitability rather than realised introduction or establishment probability. As a result, the network metrics should be interpreted as indicators of relative, not absolute, importance among islands.

## Troubleshooting

### Problem 1

Errors occur when calculating path length because some routes have trip frequency = 0 (related to step 2).

### Potential solution

• Treat routes with frequency = 0 as absent edges. Remove these edges before graph construction or before creating the distance/cost attribute. Then, create a distance variable only for edges with frequency > 0, for example:

cost=1/frequency

• Alternatively, retain all edges by creating a distance variable that accounts for zero-frequency routes, for example:

$$\text{cost}=1/\left(\text{frequency}+1e-4\right)\text{.}$$

### Problem 2

Community detection using find_partition() returns fewer nodes than are present in the network, preventing direct assignment of node names to communities and compromising modularity calculations (related to steps 6 & 15).

### Potential solution

This issue is potentially caused by fully disconnected nodes (i.e., nodes with no incoming or outgoing edges). To address this, exclude these nodes from the graph used for community detection and report them separately as isolated nodes.

## Resource availability

### Lead contact

Further information and requests for resources should be directed to and will be fulfilled by the lead contact, Marnie L. Campbell (marnie.campbell@abdn.in).

### Technical contact

Technical questions on executing this protocol should be directed to and will be answered by the technical contact, Chi T.U. Le (lethaiuyenchi1028@gmail.com).

### Materials availability

Curated vessel itineraries, SST summaries, and derived datasets for the Galapagos case study will be made available upon request.

### Data and code availability

• The data reported in the associated study are available from the lead contact upon request.
• The R code used for the analysis in the case study is provided in section “Data S1” of the supplemental information in Campbell et al.^1^

## Acknowledgments

This research received no specific funding. We acknowledge the hospitality, vital discussions, and thank the Charles Darwin Foundation, Galápagos Conservancy, Galápagos National Park Directorate (GNPD), the Galápagos Biosecurity Agency (ABG), the Ecuadorian Navy (DIRNEA), and the Navy’s Oceanographic Institute (INOCAR) for sharing their insights and biosecurity knowledge over the 10-year progress of the research. We particularly thank I. Keith, C. Causton and V. Toral (Charles Darwin Foundation), T. Dawson (University of Dundee), and K. Collins (University of Southampton) for many insightful discussions.

## Author contributions

M.L.C. conceptualized the research idea. M.L.C. and C.L.H. collected and collated the data. All authors were involved in the formal analysis and data interpretation. C.T.U.L. wrote the original draft. All authors were involved in review and editing.

## Declaration of interests

The authors declare no competing interests.

## Declaration of generative AI and AI-assisted technologies in the writing process

Microsoft Copilot (AI) was used to assist with correcting grammar and syntax errors. The manuscript was written and edited by the authors, with Copilot employed in the final stages to ensure language accuracy prior to submission. After using this tool/service, the authors reviewed and edited the content as needed and take full responsibility for the content of the published article.
